# P25. Access to diagnostics: a bottleneck for immunotherapeutics development - case example of MAGE-A3 cancer immunotherapeutic

**DOI:** 10.1186/2051-1426-2-S2-P16

**Published:** 2014-03-12

**Authors:** K Lykopoulos, P Collins, M Hall, R Ehness, J Barth, J Louahed

**Affiliations:** 1 GSK, London, UK; 2 GSK Vaccines, Rixensart, Belgium; 3 GSK, Munich, Germany

## 

Precision medicine with modern immunotherapeutics often requires pre-therapeutic access to novel and sometimes complex diagnostic tests. Test development and access to tests including reimbursement often lacks behind medicine development and availability of medicines, potentially creating a gap for physicians to administer and patients to receive an optimised treatment regime. Pharmaceutical companies are challenged by the fact of having to co-develop a diagnostic method, a process which many of them are not accustomed to. In addition, many healthcare systems have no way of allowing in parallel evaluation of diagnostic test and medicine with a view to reimbursement.

This presentation illustrates approaches using the developmental history of the MAGE-A3 cancer immunotherapeutic to both address the developmental hurdle through innovative models and partnerships, as well as the market access hurdle through appropriate market understanding and evidence generation.

Insights and solutions of pre-clinical, clinical and market research/insight will be presented.

